# Surgery

**Published:** 1895-10

**Authors:** 


### *SELECTIONS AND ABSTRACTS.*

---

### SURGERY.

Edited by Floyd W. McRae, M.D.

### Fistula in Ano.

Bacon, Joseph B., Chicago. (*Northwestern Medical Journal.*)

There were many good reasons, before the antiseptic treatment of wounds, why so many fistulas could not be successfully handled, but the main reasons are:

First, the general practitioner has failed to realize that all fistulas are the result of abscesses, and that the fistulous tract is but the collapsed abscess wall, and that naturally there would be a tendency for the pus to have burrowed in most cases in various directions, leaving this collapsed abscess wall complicated by numerous channels radiating from the central canal.

Second, that until recently the student was not required to make careful dissections of the pelvic fascias, perineal and anal region, thus leaving him, when in practice, with a vague idea of the direction the pus from an abscess in these regions would likely take in accordance with the anatomical arrangements of the tissues. Repeated dissections only will give one an idea of the possibilities of the complications that may attend these abscesses, and also show how difficult the diagnosis and treatment may become.

Usually the abscesses will occur either subcutaneously, submucously, or in the ischio-rectal fossæ, and still rarer, yet most difficult when it occurs in the superior peri-rectal space. The various types of resulting fistulas will depend upon where and in how many places these abscesses have gained an outlet. While the division of some authors into first, blind internal, complete and incomplete; second, blind external, complete and incomplete; third, complete internal; fourth, complete external, will describe the greater number of fistulas one meets, there may be various complications with those that would make a more extensive classification possible.

The etiology of abscesses here may be the same as in other locations. Likewise, the symptoms do not differ from any other source of irritation around the anal region, except when there is an outlet for the pus secretion in such a location as to attract attention from its constant escape.

There are no secret remedies for the treatment of fistulæ. The same surgical principles apply here as well as elsewhere. Absolute cleanliness and disinfection of the tract with peroxide of hydrogen, listerine, bichloride solutions, and the various antiseptics, together with keeping the fistulous tract patulous, will cure a great many of these cases. Allingham's method of keeping the external opening dilated is an excellent one, and simply consists in inserting into the opening a bone collar button that has had a hole drilled through it lengthwise. Curetting or cauterizing the tract sufficiently to set up inflammatory conditions will hasten granulation and repair.

These palliative measures should only be used for a short time, when, if the tract has not healed, radical measures for laying open the tract must be taken, for long-continued suppuration always tends to amyloid degeneration of the viscera, with general constitutional disturbances.

In doing a radical operation for fistula there are some important points to be observed:

1. Never sever the sphincters at more than one place at the same operation, no matter what the complications may be, otherwise incontinence is sure to follow.

2. Unless all the channels are followed up and laid open the operation will fail of its purpose.

3. Fistula resulting from tubercular abscess must not be operated upon if there is sufficient tissue destruction of lung to produce hectic fever, sweats, etc., unless the fistula is causing severe painful spasms of the sphincters, then it should be divided at any stage.

4. After laying the fistulous tract open the wound must be made to heal from the bottom, and as the cutaneous or mucous side of the wound is better nourished it will throw out a more healthy granulation that tends to bridge over and close the slower granular surface at the bottom, thus leaving a fistula remaining.

5. When the fistulous tract is not too complicated it should be dissected out entire and the wound brought together, beginning at the bottom with continuous catgut sutures, and approximating the surfaces in successive layers until the whole wound is closed.

All operations about the anus, although theoretically septic, should be done with the same care and dressed the same as for a laparotomy, then more of them will heal without formation of pus.

The general prejudice among the laity against having fistulæ cured has been taught them by ignorant physicians, and to disabuse their minds of this idea will take time and concerted action of the profession. It is a common thing to see patients in the hospitals whose viscera have undergone amyloid degeneration, and they have become hopelessly wrecked by a long-continued septic absorption from a fistulous tract that a minor operation would have relieved at its commencement.—*Mathews Medical Quarterly.*

---

### SOME CASES OF ACUTE INTESTINAL OBSTRUCTION WITH DEDUCTIONS.

Cartledge, A. Morgan. (*International Medical Magazine,* May, 1895.)

The author reports six cases of intestinal obstruction tending to confirm the accepted belief of the hopeless nature of this condition unless treated by early laparotomy.

The cause of obstruction in the first case was strangulated hernia. In the second case there was volvulus of the descending colon with abscess. The third was a case of intussusception; whilst the fourth was a case of a band-like constriction, produced by the adhesion of two folds of the mesentery forming a mesenteric pocket. In the fifth case a patient walked across a room on the third day after laparotomy for double pyosalpinx. She was seized with agonizing pain in the abdomen. At the operation several coils of intestine were found adherent, a tightly constricted coil between the margins of the left broad ligament, and a coil stretched across and adherent to the raw surface behind the uterus. In the sixth case there was strangulation of a loop of small intestine in a snare formed by adhesion of a point of omentum to the stump of removed tube after laparotomy for double pyosalpinx.

In all these cases laparotomy was performed, three recovering and three having a fatal result.

Dr. Cartledge considers that his cases help to prove that "the large death-rate from operation in acute intestinal obstruction does not come from lack of adequate surgical methods to deal with every phase of the lesion encountered, but from the late time at which the relief is supplied." He also points out how very serious conditions may be masked by the use of opium, and suggests that this drug might be withheld where obstruction is at all suspected till a diagnosis can be made.

"The burning question now is to educate men to know that action to be successful must be quick." A medical man need not waste his time in trying to determine whether it be a probable intussusception, volvulus, band, or diverticulum which has caused obstruction. Operation is the most reliable way of finding out. The cardinal points in avoiding a fatal delay and making an early diagnosis are to be found in sudden abdominal pain, a rapidly accelerating pulse, the vomiting of much more fluid in a given time than that taken by the mouth, the green-tinged character of this fluid, the anxious expression of countenance where opium has not been given, and the fact that although enemata may be stained by colon contents, there is no expulsive movement of the bowel and no passage of gas.

"The pulse is the sole indicator of the acuity of cause and status of the patient in intestinal obstruction. If a patient is seen at 2 P. M., and the pulse is found to be 110, to prescribe for such a patient with the determination to operate the following day if he is no better, usually means that while the physician is taking his much-needed rest that night the patient is passing forever beyond the realm of operative relief. The next morning the physician will probably be much surprised and chagrined to find the pulse 140, and the patient moribund."—*Med. Chronicle.*

